# Smooth transportation of liquid metal droplets in a microchannel as detected by a serially arranged capacitive device

**DOI:** 10.1038/s41598-021-86394-w

**Published:** 2021-03-29

**Authors:** Satoshi Konishi, Yugo Kakehi, Fuminari Mori, Shinji Bono

**Affiliations:** 1 Department of Mechanical Engineering, College of Science and Engineering, Ritsumeikan University, Kusatsu, 525-8577 Japan; 2 Graduate Course of Science and Engineering, Ritsumeikan University, Kusatsu, 525-8577 Japan; 3 Ritsumeikan Global Innovation Research Organization, Ritsumeikan University, Kusatsu, 525-8577 Japan

**Keywords:** Materials for devices, Fluidics, Wetting, Soft materials, Fluids

## Abstract

Gallium alloy liquid metals (Galinstan) possessing fluidity, electric conductivity, and low toxicity are attractive for use in flexible devices and microfluidic devices. However, the oxide skin of Galinstan in the atmosphere adheres to the microchannel surface, preventing the transportation of Galinstan in the channel. To tackle the problem of the adhesion of Galinstan to microchannel, we introduced liquid with Galinstan into a channel with a diameter of 1000 μm. Then, we found that the cylindrical shape of the channel enabled smooth transportation of Galinstan independently of both the liquid and the channel material. The liquid introduced with Galinstan not only prevents adhesion but also improves the spatial controllability of Galinstan in the channel. We can control the position of Galinstan with 100 μm resolution using highly viscous (> 10 cSt) liquid. In addition, we combined the microchannel with patterned electrodes, fabricating a serially arranged capacitive device. The local capacitance detected by the patterned electrodes changed by more than 6% via the smooth transportation of Galinstan. The analysis results based on an equivalent circuit quantitatively agree with our experimental results. We can modulate the serially arranged capacitors using the smooth transportation of Galinstan in the channel.

## Introduction

Liquid metal (LM) has both fluidity and high electrical conductivity and is an appropriate material for electrical wiring and sensors in flexible devices^1–3^. Since LM is soft and has a smooth shape, it is easily introduced into microchannels, indicating that it has promising microfluidic device applications, such as in switches and valves^4^. A well-known LM is mercury, which shows fluidity and high electric conductivity at room temperature. Hence, mercury has been used broadly in electric switches, diodes, and pressure gauges. However, because of the large burden on the environment and the high toxicity of mercury, its usage has recently been avoided^5,6^. Gallium alloy-LM (Galinstan) is a fascinating alternative to mercury because Galinstan shows low toxicity as well as fluidity and electrical conductivity^7,8^. Galinstan is applicable to the flexible devices as well as mercury. In addition, the fluidity of Galinstan is useful in the photocatalysis application^9^ and the conductive patterning application^10^.

The application of Galinstan to microdevices presents the following challenges: in the atmosphere, gallium at the surface is oxidized, and as a result, a thin (1–3 nm) oxide skin is spontaneously formed^2,11^. The oxide skin is adhesive to surfaces, which inhibits Galinstan from being transported smoothly in the microchannel and causes disconnection and deterioration of flexible devices^12^. In addition, the gallium oxide skin is an electric insulator (~ a few MΩ)^13^. Thus, the application of Galinstan based on its electrical conductivity is difficult without nontrivial treatment to remove the oxide skin.

By chemical surface treatment with a strong acid or base, the oxide skin can be removed, and thus both the fluidity and the electric conductivity can be maintained^14,15^. However, Galinstan in the atmosphere continues to be oxidized spontaneously. To take advantage of the promising properties of Galinstan with chemical treatment, continuous chemical treatment is necessary; that is, microdevices must contain a strong acid or base with Galinstan. Therefore, the chemical treatment requirement is a disadvantage in terms of practical device applications.

Recently, it was reported that liquid, such as water, introduced with Galinstan into microchannels can prevent the oxide skin from adhering to the surfaces of microfluidic devices^16^. The fluidity of Galinstan dispersed in liquid can be maintained without harsh chemicals, which is an advantage over chemical treatment. However, the mechanism for preventing gallium oxide from adhering is unclear. Because of the fluidity, Galinstan is appropriate material for the microfluidic application. Thus, the condition to transport Galinstan through the microchannel must be revealed. In principle, with this method, it is impossible to remove the oxide skin, making applications based on electric resistance difficult without additional treatment. Therefore, it is important to use electrical properties that can be detected without direct contact^17,18^. For example, capacitance is promising candidate because the position of Galinstan in the microchannel should couple with the local capacitance which can be measured indirectly.

In this paper, we solved the problem of the adhesion of Galinstan to microchannel, focusing on the geometry of and materials in the microfluidic devices. After introducing Galinstan into the microchannel, we applied pressure to the Galinstan. To control the position of the Galinstan in the microchannel, we prevented Galinstan from adhering to the surface of the channel and stably transported Galinstan while keeping its shape intact. In particular, we quantitatively estimated the reproductivity, which is an important parameter for the spatial control of Galinstan. Then, we fabricated a serially arranged capacitive device in which Galinstan are transported smoothly. We modulated the local capacitance using the smooth transportation of Galinstan. We discussed the local capacitive modulation and the spatial resolution of Galinstan droplet in the microchannel.

## Results

**Figure 1:**
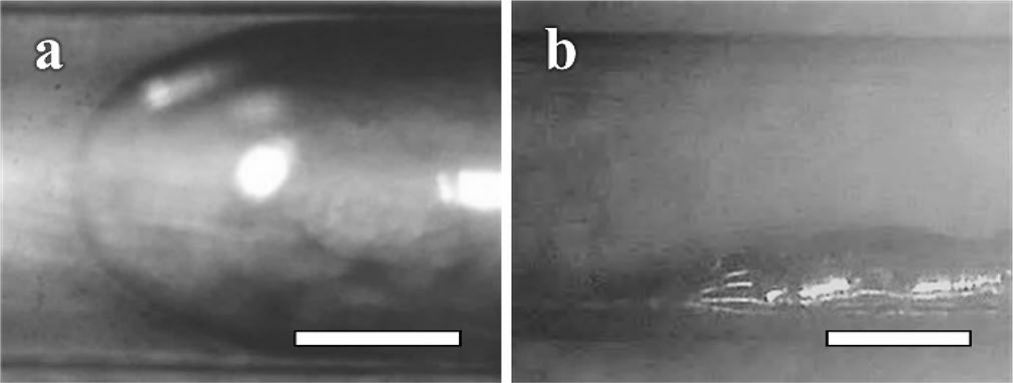
Microscope images of the Galinstan and water. (**a**) Microscope image in the cylidrical microchannel and (**b**) Microscope image in the square pole-shaped channel. The scale bars indicate 250 μm.

Figure 1a shows a microscopic image of a Galinstan and water in the cylindrical microchannel (diameter: ϕ = 600 μm). The Galinstan did not adhere to the surface of the channel and maintained a smooth, intact shape. While pressure was applied to the inlet, the moving Galinstan never adhered to the channel surface. Then, to investigate the effect of the channel material of the microfluidic device, we introduced Galinstan into cylindrical channels composed of Teflon (ϕ = 1 mm and 0.8 mm), vinyl chloride (ϕ = 1 mm), and glass (ϕ = 0.6 mm). As a result, we confirmed that the Galinstan is transported smoothly without adhesion to the surface of the channels. In addition, we observed the transportation of Galinstan with silicone oil, Fluorinert, or vegetable oil, whose chemical properties differ from each other. As a result, we confirmed that the oxide skin of Galinstan does not adhere to the channel surface independently of the liquid used. These results suggest that the materials that make up the channel and the liquid do not affect the transportation of Galinstan.

Figure 1b shows a microscope image of the Galinstan and water in a square pole-shaped channel. Since the Galinstan adhered to the channel surface just its injection with water, the Galinstan does not show fluidity in the square pole-shaped channel. Next, we fabricated a hybrid microchannel composed of glass and polydimethylsiloxane (PDMS), which is a typical combination in fluid microelectromechanical systems (MEMSs)^19,20^. However, the Galinstan in the square pole-shaped hybrid microchannel adhered to the channel surface. Therefore, the cylindrical shape of the microchannel is important for the smooth transportation of Galinstan.

**Figure 2:**
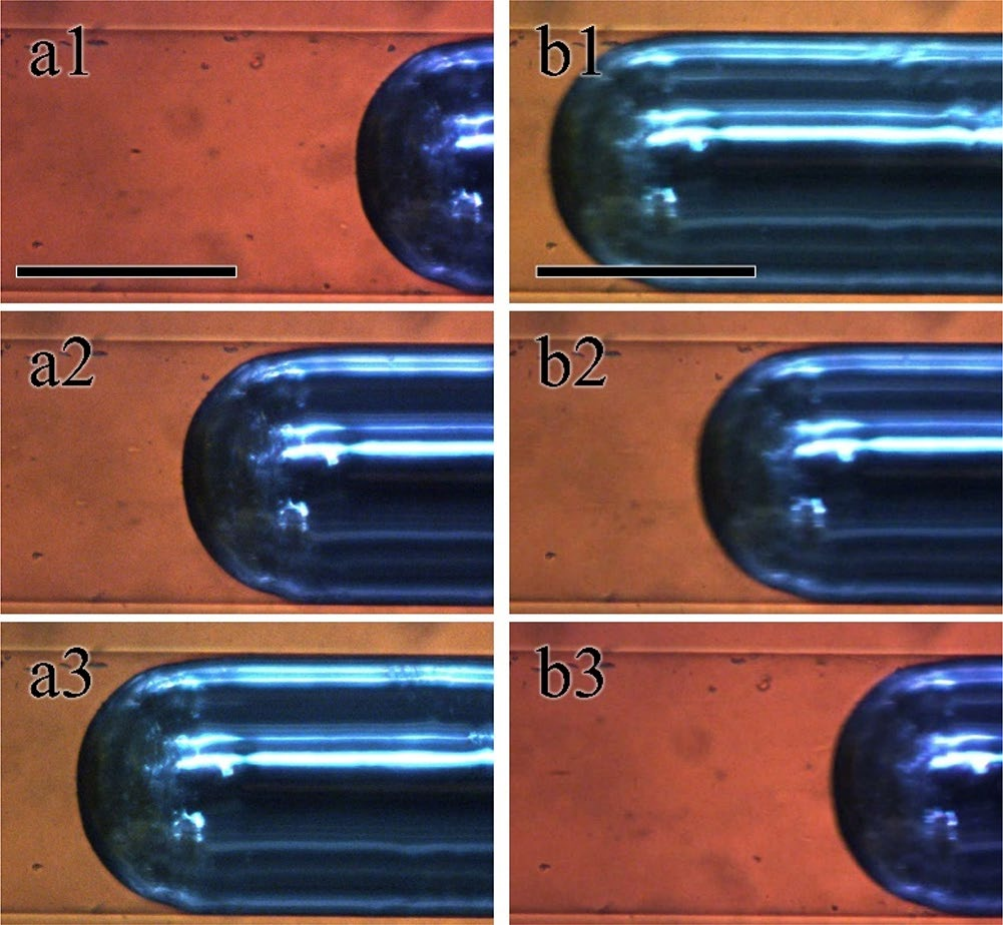
Optical microscopic images of Galinstan droplet in microchannel. (**a1**–**a3**) Galinstan droplet in silicone oil under positive pressure. The snapshots were taken every 5 s. (**b1**–**b3**) Optical microscopy images of Galinstan in silicone oil under negative pressure. Snapshots were taken every 1 s. The scale bars indicate 500 μm.

Since the spatial resolution of the Galinstan in the microchannel is directly related to the detection resolution of devices, we focused on the spatial control of Galinstan. We sealed the outlet and repeatedly applied positive (50 kPa) and negative pressure on Galinstan in the microchannel. After 50 compression processes, we measured the spatial deviation from the initial position. As a result, the deviation of Galinstan in water was larger than 2 mm. This is caused by the time lag between the switching of the pressure direction and that of the transporting direction of Galinstan due to the inertia of Galinstan in low viscosity liquid. Then, we used highly viscous liquids, that is, vegetable or silicone oil, instead of water. Figure 2(a1–a3) shows optical microscopy images of Galinstan and silicone oil under positive pressure. Although the kinetic viscosity of silicone oil is much higher than that of water, the Galinstan is transported smoothly. In contrast, the Galinstan is transported in an inverse direction under negative pressure, as shown in Fig. 2(b1–b3). We summarized the liquids used and the kinetic viscosity and deviation of Galinstan from the initial position in Table 1. When Galinstan disperse in a highly viscous liquid, the deviation becomes smaller than the observation resolution of the microscope (< 100 μm).

**Table 1:** Kinetic viscosity and the deviation from the initial position after 50 compression processes.

Material	Kinetic viscosity (25 °C) (cSt)	Deviation (mm)
Water	0.893	> 2
Vegetable oil	10	< 0.1
Silicon oil	100	< 0.1

**Figure 3:**
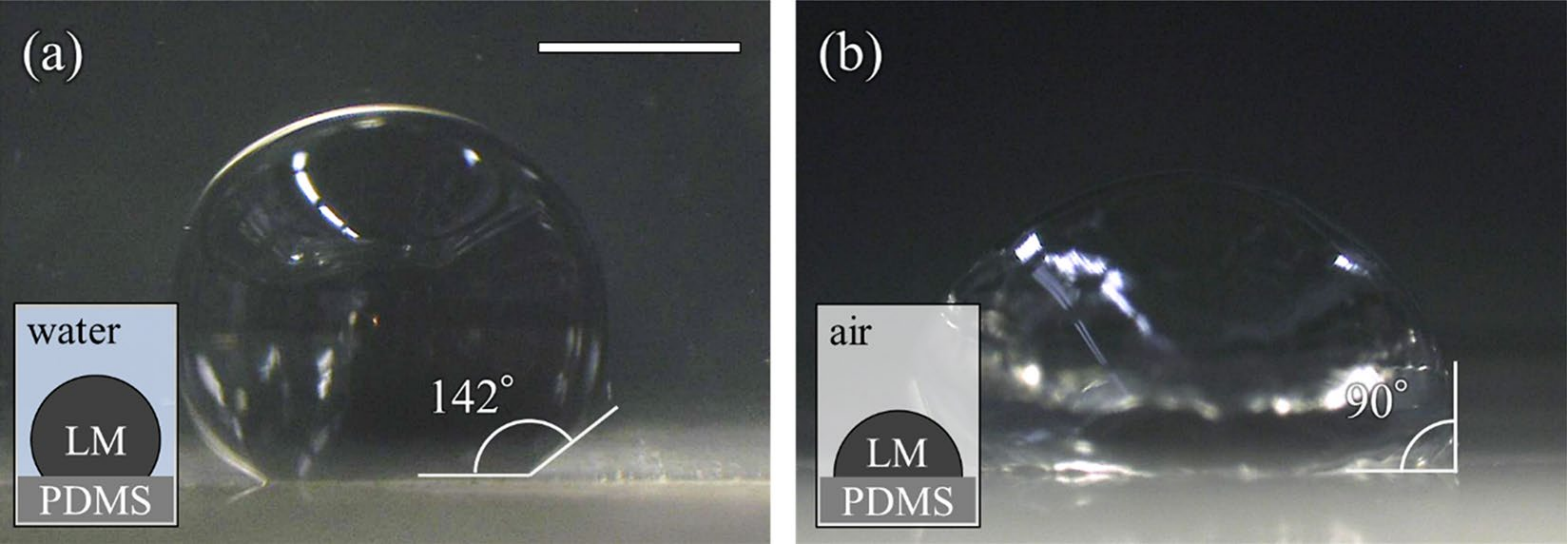
Contact angle of Galinstan droplet on PDMS-film. (**a**) Microscope images of the Galinstan droplet under water environment, and (**b**) under gas environment. The scale bar indicated 1 mm. The schematic insets show the experimental setup.

To estimate the adhesion of Galinstan to the microchannel composed of PDMS, we measured the contact angle on the flat PDMS-film. We put the flat PDMS-film on the bottom of a container and filled the container with water. Then, we dropped 4 μl of Galinstan on into water. Sideview of Galinstan droplet was obtained by the microscope. Figure 3a shows the microscope image of the Galinstan droplet under water environment. Contact angle was estimated at 142°, which indicates that Galinstan droplet dewets PDMS. We removed water and observed Galinstan droplets under air environment. The contact angle decreases to 90°, which indicates that Galinstan adhere to PDMS-film. Therefore, we concluded that liquid prevents the Galinstan droplet from adhering to the microchannel.

**Figure 4:**
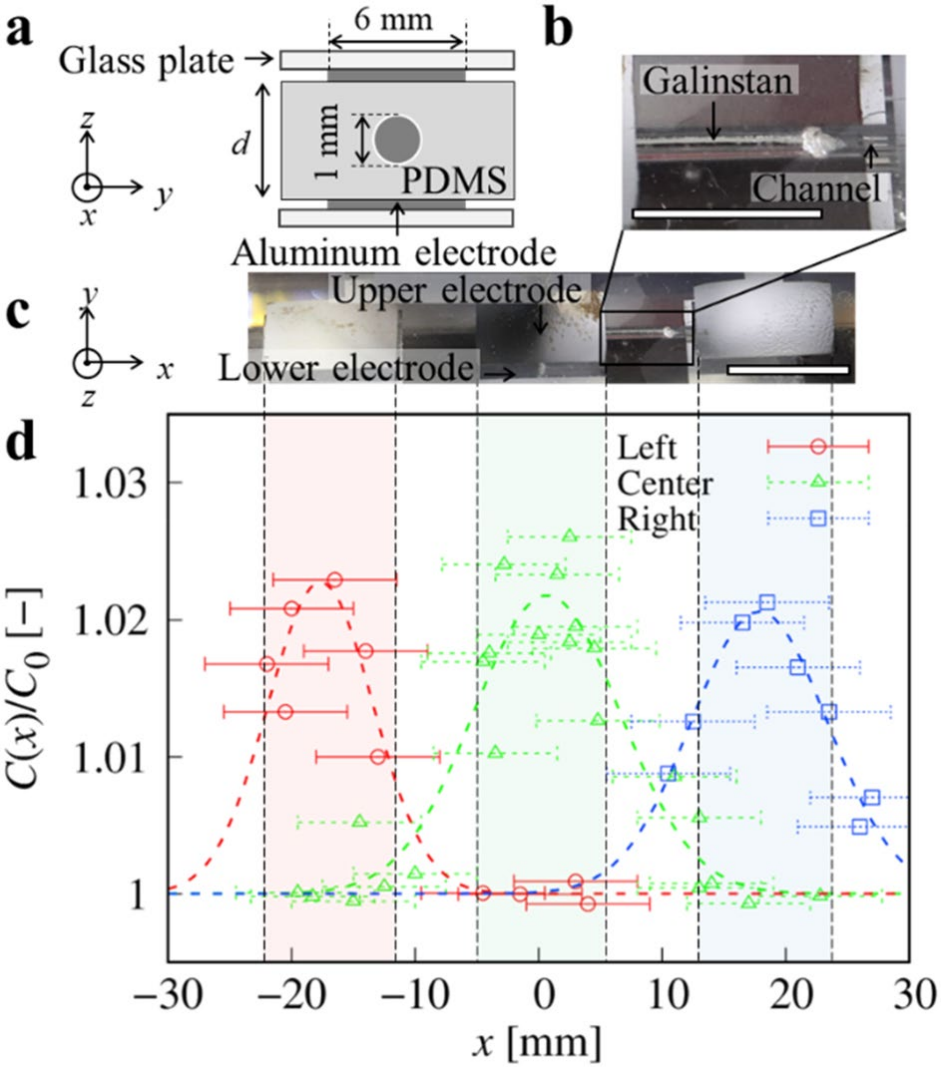
The serially capacitive device. (**a**) Schematic cross section perpendicular to the microchannel. (**b**) An enlarged photograph of Galinstan in the microfluidic channel. The diameter of the channel is 1.0 mm. The scale bar indicates 5 mm. (**c**) A perspective photograph of the serially arranged capacitive device. The channel was sandwiched by upper and lower aluminum electrodes at three points, that is, at the left, center, and right. The scale bar indicates 10 mm. (**d**) Normalized capacitance *C*(*x*)/*C*_0_ as a function of the position of Galinstan, *x*. Red, green, and blue markers indicate the capacitance measured at the left, center, and right electrodes, respectively. We provided a visual guide to indicate the position of the electrodes.

To fabricate a serially arranged capacitive device using the smooth transportation of Galinstan droplets, we investigated the relation between capacitance and the position of Galinstan in a microchannel. Then, we sandwiched the microchannel between patterned electrodes and investigated the local capacitance of Galinstan introduced in the channel. Figure 4a shows a schematic cross section of the serially arranged capacitive device. The diameter of the channel was 1.0 mm. We defined the thickness of the channel composed of PDMS as *d* and used a channel with *d* = 2.3 mm. The Galinstan in the microchannel is enlarged in Fig. 4b. We connected the syringe pump to the inlet to control the position of Galinstan by pressure. Figure 4c shows a photograph of the serially arranged capacitive device. The microchannel was sandwiched between two glass substrates, on which we patterned three pairs of aluminum electrodes. Via capacitance measurements, a pair of electrodes is connected to an inductance–capacitance–resistance (LCR) meter. We measured *C*_0_ and *C*(*x*), where *C*_0_ and *C*(*x*) are the capacitance without Galinstan, i.e., the channel is occupied by pure silicone oil, and the capacitance as a function of the position of Galinstan, *x* (we took the origin at the center of our device), respectively. *C*_0_ was found to be 3–4 pF. Since the dielectric constant of silicone oil is almost the same as that of PDMS, and *C*_0_ = ε*S*/*d*, substituting the experimental values of ε ~ 3 × 10^−11^ F/m, *S* ~ 6 × 10^−5^ m^2^, and *d* ~ 2 × 10^−3^ m, we obtain *C*_0_ ~ 1 pF, which agrees with the experimental value.

The red, green, and blue markers in Fig. 4c indicate the capacitance measured at the left, center, and right electrodes, respectively. Here, we normalized *C*(*x*) as *C*(*x*)/*C*_0_ to neglect the difference between the shapes of those electrodes. When Galinstan reach a position between the electrodes, the normalized capacitance *C*(*x*)/*C*_0_ takes the maximum value. In contrast, when the Galinstan are far from the electrodes, *C*(*x*)/*C*_0_ takes a constant minimum value. The change in *C*(*x*)/*C*_0_ is larger than 2%, which indicates that we can modulate the serially arranged capacitors using the smooth transportation of Galinstan in the microchannel.

**Figure 5:**
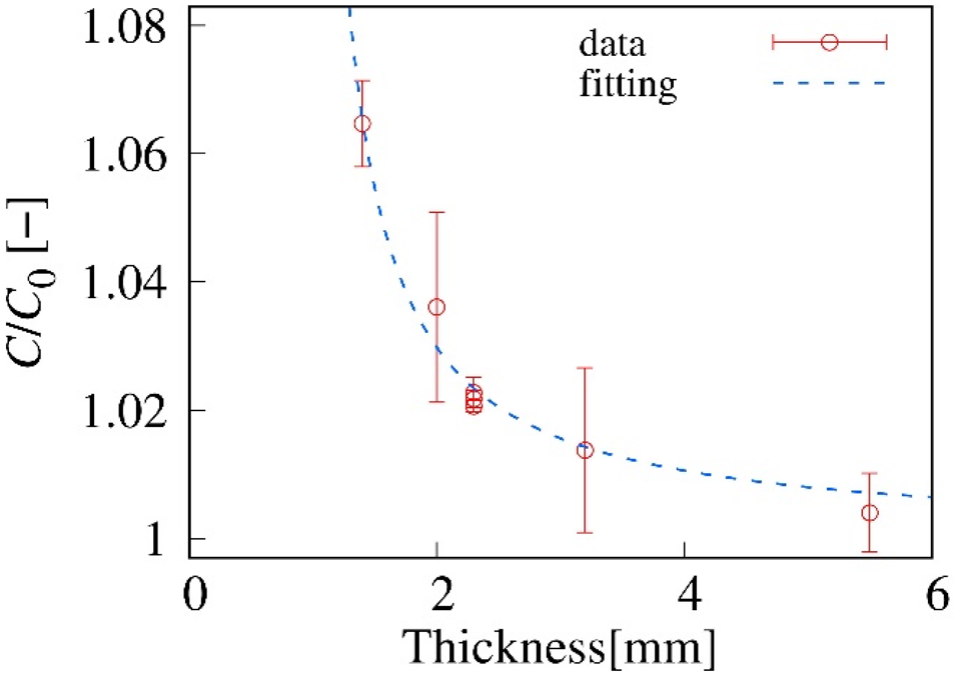
Dependence of the capacitance of Galinstan on the PDMS thickness. Red and blue markers indicate the experimental results measured by uniform electrodes and the maximum change estimated from the results shown in Fig. 4(c), respectively. The diameter of the channel is 1 mm. The green line is the best fit curve obtained using Eq. (5a).

One of the important factors that determined the change in the capacitance is the volume ratio of Galinstan to PDMS. We fabricated microfluidic devices composed of PDMS with a thickness of 1.4–5.5 mm. The thickness of PDMS must be greater than 1 mm because the diameter of the channel is 1 mm. Then, we sandwiched the microfluidic device between uniform electrode substrates and measured *C*_0_ and *C*, where *C* is the capacitance under the condition that the channel is occupied by Galinstan. Figure 5 shows the PDMS thickness dependence of the normalized capacitance *C*/*C*_0_. Red markers indicate the *C*/*C*_0_ measured by uniform electrodes. The blue markers indicate the maximum change in *C*(*x*)/*C*_0_ estimated from the results shown in Fig. 4c. When the thickness of a device is 1.4 mm, the *C*/*C*_0_ is larger than 6%. In addition, *C*/*C*_0_ monotonically decreases with increasing thickness.

## Discussion

### Smooth transportation of Galinstan

In the cylindrical channel, the Galinstan is subjected to a uniform force from the channel. As a result, the liquid layer is uniformly inserted between Galinstan and the channel, preventing Galinstan from adhering to the channel. In contrast, Galinstan in the square pole-shaped channel are under heterogeneous forces from the channel. For example, the force from the flat part of the channel is different from that of the edge. Since Galinstan are forced against the surface of the channel by heterogeneous forces, the oxide skin adheres to the channel. Therefore, to smoothly transport Galinstan through the microchannel, the shape of the cross section must be a circle, independent of the liquid and channel material.

### Change in the capacitance with and without Galinstan

Since silicone oil in the channel is replaced by Galinstan via smooth transportation, the local capacitance increases. To quantitatively analyze the change in the capacitance, we constructed an equivalent circuit of our device with two approximations: (i) the distribution of electric charge in the parallel plane to the electrodes is uniform, and (ii) the capacitance out of the device is negligible because the capacitances of PDMS and the channel are larger than that of air.

**Figure 6:**
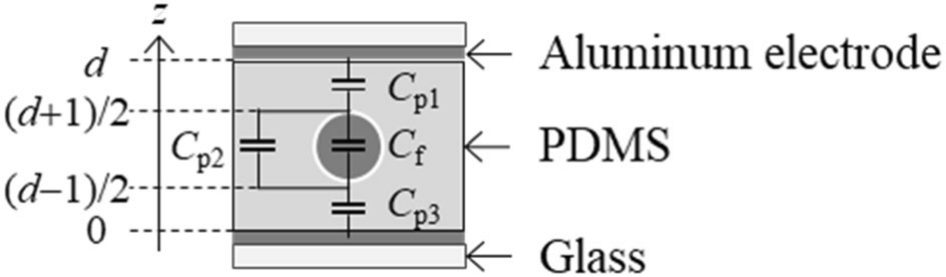
Schematic cross section of the channel and the equivalent circuit.

Figure 6 shows a schematic cross section of the channel in PDMS with thickness *d* and a width of 6 mm. We superimposed the equivalent circuit on the cross section, as shown in Fig. 6. The *z*-axis is perpendicular to the electrodes. We regarded PDMS in the ranges of (*d* + 1)/2 < *z* < *d* and 0 < z < (*d *− 1)/2 as capacitors with capacitance,

1 $$ C_{p1} = C_{p3} = \varepsilon_{p} S\frac{2}{d - 1} $$

where ε_p_ and *S* are the dielectric constant of PDMS and the electrode area, respectively. We regarded the channel and PDMS in the range of (*d *− 1)/2 < *z* < (*d* + 1)/2 as two parallel capacitors because we neglected the heterogeneity of electric charges in the perpendicular plane to the *z*-axis. The capacitance of PDMS in the range of (*d *− 1)/2 < *z* < (*d* + 1)/2 is given as

2 $$ C_{p2} = \varepsilon_{p} \left( {1 - \frac{\lambda }{6}} \right)S $$

where λ is the geometric parameter that corresponds to the area ratio of the channel to PDMS. For geometrical reasons, λ < 1. Since silicone oil is mainly composed of PDMS, we regarded the dielectric constant of silicone oil as ε_p_. Then, the capacitance of the channel occupied by silicone oil, *C*_SO_, and that occupied by Galinstan, *C*_LM_, are denoted as

3 $$ C_{f} = \left\{ {\begin{array}{*{20}c} {C_{SO} = \varepsilon_{p}  \frac{\lambda }{6} S \,({\text{silicon oil}})} \\ {C_{LM} = \alpha C_{SO} \,({\text{Galinstan}})} \\ \end{array} } \right. $$

where α is the ratio of the capacitance with Galinstan to that with silicone oil. Equations (1–3) indicate that the total capacitance of the equivalent circuit is given as

4 $$ \frac{1}{C} = \frac{1}{{C_{p1} }} + \frac{1}{{C_{f} + C_{p2} }} + \frac{1}{{C_{p3} }} $$

The normalized capacitance *C*/*C*_0_ is given as

5a $$ \frac{C}{{C_{0} }} = \frac{d}{d - \delta (\alpha , \lambda )} $$

and

5b $$ \delta (\alpha , \lambda ) = \frac{\lambda (\alpha - 1)}{{6 +\lambda (\alpha - 1)}} $$

Equation (5a) indicates that the electrode gap *d* is modified as *d *− δ by replacing silicone oil with Galinstan in the channel. We fitted the experimental results with Eq. (5a), where δ is the only fitting parameter, and showed the best fit curve in Fig. 5 as the green line. Our model based on the equivalent circuit agrees approximately with the experimental results. A slight quantitative disagreement is thought to be caused by our approximations, where we neglected the electric heterogeneity in the parallel plane to the electrodes. The fitting result indicates that δ  ~ 70 μm, and α = 1.5—6. This result suggests that the capacitance increases 1.5–6 times by introducing Galinstan into the channel.

### Motion and position detection of transported Galinstan droplets in a microchannel

We succeeded in changing the capacitance of serially arranged capacitors using the smooth transportation of Galinstan. In the serially arranged capacitive device, the normalized capacitance *C*(*x*)/*C*_0_ depends on the relative positions of Galinstan and the electrodes. When the Galinstan is far from the electrodes, *C*(*x*)/*C*_0_ takes a constant minimum value. After intruding into the channel sandwiched by the electrodes, *C*(*x*)/*C*_0_ starts increasing. *C*(*x*)/*C*_0_ eventually reaches a maximum value at the position where the center of the Galinstan corresponds with that of the electrodes. These results indicate that *C*(*x*)/*C*_0_ monotonously depends on the volume ratio of Galinstan in the channel between the electrodes.

For microfabrication, (1) the length of the Galinstan droplets and the width of the electrodes should correspond with each other to improve the spatial resolution of the Galinstan near the center of the electrodes. Under the condition that the length of Galinstan is not equal to the width of electrodes, the spatial deviation of Galinstan from the center of the electrodes is not accompanied by a change in *C*(*x*)/*C*_0_ because the volume fraction of Galinstan is invariant near the center of the electrodes. (2) The length of Galinstan should be longer than the interval between pairs of electrodes. Unless a part of Galinstan overlaps with electrodes, the spatial change in Galinstan does not cause a change in *C*(*x*)/*C*_0_. Since the position of Galinstan droplet in the channel whose diameter is larger than the size of the Galinstan droplet is unstable along the depth direction, Galinstan must be larger than the diameter of the channel. Thus, we found that the limitation of the microfabrication is ~ 1 pattern/mm. Since the occupation volume of Galinstan in the microchannel is much smaller than that of the liquid, the viscous dissipation in Galinstan is negligible. Here, we assumed that the flow field in the microchannel is Poiseuille flow. The volumetric flow rate is given as

6 $$ Q \sim \frac{\pi }{8\gamma } a^{4} \frac{P}{l}, $$

where *P*, *a*, *l*, and γ are the pressure difference between inlet and outlet, the radius of the microchannel, the length of the microchannel, and viscous coefficient, respectively. Signal frequency is determined as *v*/*d*, where *v* and *d* are the velocity of Galinstan droplet and electrode interval, respectively. Substituting the experimental values of *P* ~ 30 kPa, *a* ~ 500 μm, *l* ~ 500 mm, and γ ~ 0.1 Pa/s, we obtained *Q* ~ 15 mm^3^/s and the average transportation velocity of Galinstan droplet ~ 10–20 mm/s. Because of the limitation of microfabrication (1/*d* ~ 1 pattern/mm), the signal frequency was estimated as ~ 10 Hz.

We modulated serially arranged capacitors using the smooth transportation of Galinstan droplets, which conversely indicates that we can sense the position of Galinstan in the channel from the change in capacitance. The change in the capacitance is 10^−2^ pF/mm. Taking into account the resolution of the LCR meter (10^−2^ ~ 10^−3^ pF), the position of Galinstan can be detected with a 100 μm resolution. The deviation after the transport process was repeated was found to be approximately 100 μm. Thus, we can sense the position of Galinstan based on serially arranged capacitors with a comparable resolution with spatial controllability.

## Method

### Observation of Galinstan in a microchannel

We introduced Galinstan (eutectic gallium indium stannum, Zairyo-ya.com) and liquid into a microchannel composed of PDMS (DuPont Toray Specialty Materials K.K.). We used water, vegetable oil (Kadoya Sesame Mill inc.), Fluorinert (3 M), and silicone oil (MOMENTIVE) as the liquids. One side of the through-hole of the PDMS microchannel was an inlet, and the other side was an outlet. We fabricated two kinds of channels, that is, a cylindrical channel and a square pole-shaped channel. The diameter of the cylindrical channel ϕ was 600 μm–1 mm, and the dimensions of the square pole channel were 500 μm × 500 μm. For observation of Galinstan in the microchannel, we used a microscope (DIGITAL MICROSCOPE VHX-500F, KEYENCE) and a transmission optical microscope (MX9430, MEJI TECHNO). All observation and experiments in this paper were performed at room temperature.

### Position control of Galinstan in microchannel

We introduced Galinstan and liquid from the inlet. Then, we sealed the outlet with epoxy glue, where a small amount of air between the liquid and outlet was maintained. To transport Galinstan from the inlet to the outlet, we applied 30 kPa pressure to the inlet for 5 s. For transportation in the backward direction, we removed the pressure for 5 s.

### Position of Galinstan and serially arranged capacitors

A PDMS microchannel with ϕ = 1 mm and diameter *d* was sandwiched by glass plates whose surface was patterned with aluminum electrodes. Electrodes were connected with an LCR meter (LCR METER, KEYSIGHT). We injected Galinstan and silicone oil into the microchannel with a syringe pump. To control the position of Galinstan in the microchannel, we applied pressure to the channel. For capacitance measurements, aluminum electrodes connected to an LCR meter were applied to an AC electric field at 2 V and 500 kHz. We measured the capacitance and the position of Galinstan simultaneously.

The total capacitance of PDMS and the microchannel is approximately ε*S*/*d*, where ε, *S*, and *d* are the dielectric constant of PDMS, the electrode area, and the electrode gap, respectively. The resolution of capacitance increases with decreasing electrode gap. The minimum electrode gap is the diameter of the microchannel. For this reason, we designed an electrode gap on a similar order to the diameter of the microchannel (~ 1 mm).

We used two kinds of electrode substrates to evaluate the capacitance.

**Figure 7:**
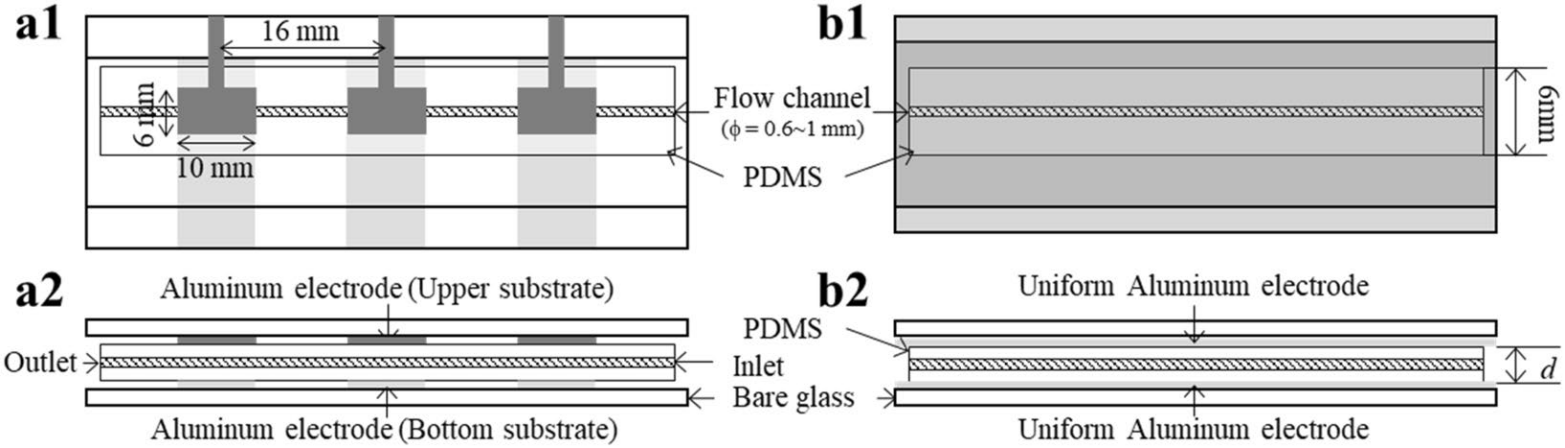
The patterned electrode and the uniform electrode. (**a1**) Schematic top view and (**a2**) schematic side view of the patterned electrode. We deposited three aluminum electrodes with dimensions of 6 mm × 10 mm on the upper glass substrate with intervals of 16 mm. We deposited three aluminum electrodes whose width was 10 mm on the lower glass substrate with an interval of 16 mm. (**b1**) Schematic top view and (**b2**) schematic side view of uniform electrodes.

1. Patterned electrodes (Fig. 7a1–2)

We patterned the upper glass substrate with three aluminum electrodes whose dimensions were 6 mm × 10 mm. The intervals of electrodes were 16 mm. The lower glass substrate was patterned with three aluminum electrodes whose width was 10 mm with a 16 mm spacing. We connected a pair of electrodes to an LCR meter to measure the local capacitance. Based on the balance between spatial and measurement resolutions, the electrode area *S* was set to 60 mm^2^.

(2) Uniform electrodes (Fig. 7 (b1-2))

We deposited uniform aluminum electrodes on glass substrates to accurately measure the capacitance. PDMS microchannels with a width of 6 mm and diameter *d* were sandwiched by uniform electrode substrates whose electrode area *S* was ~400 mm^2^. Upper and lower electrodes were connected to an LCR meter to measure the total capacitance of the fluidic device.

## Conclusion

In this paper, we overcame the problem of the adhesion of Galinstan to microchannel. We showed that Galinstan introduced into a cylindrical channel with liquid are transported smoothly independently of the liquid and the channel material because the Galinstan in the cylindrical channel is subjected to a uniform force from the channel. The position of Galinstan can be controlled with a resolution of 100 μm using highly viscous (~ 10 cSt) liquid. These results indicate that the smooth transportation of Galinstan droplets is applicable for microfluidic device with high spatial resolution. Then, we fabricated serially arranged capacitive device and measured the local capacitance indirectly. The local capacitance of the patterned electrode device can be modulated by the position of Galinstan in the channel. The prediction of our equivalent circuit model agrees with the experimental behavior, which indicates that Galinstan droplet locally increases the capacitance. These results suggest that the smooth transportation of Galinstan through a channel is applicable for the modulation of serially arranged capacitors. Moreover, we showed the possibility of applying a serially arranged capacitive device as a spatial sensor of Galinstan droplets even in a microfabrication system.

## Data Availability

All data are included in this published article.
